# Recognizing ion ligand binding sites by SMO algorithm

**DOI:** 10.1186/s12860-019-0237-9

**Published:** 2019-12-11

**Authors:** Shan Wang, Xiuzhen Hu, Zhenxing Feng, Xiaojin Zhang, Liu Liu, Kai Sun, Shuang Xu

**Affiliations:** College of Sciences, Inner Mongolia University of Technology, Hohhot, 010051 China

**Keywords:** Ion ligand, SMO algorithm, Binding site, Sequence information

## Abstract

**Background:**

In many important life activities, the execution of protein function depends on the interaction between proteins and ligands. As an important protein binding ligand, the identification of the binding site of the ion ligands plays an important role in the study of the protein function.

**Results:**

In this study, four acid radical ion ligands (NO_2_^−^,CO_3_^2−^,SO_4_^2−^,PO_4_^3−^) and ten metal ion ligands (Zn^2+^,Cu^2+^,Fe^2+^,Fe^3+^,Ca^2+^,Mg^2+^,Mn^2+^,Na^+^,K^+^,Co^2+^) are selected as the research object, and the Sequential minimal optimization (SMO) algorithm based on sequence information was proposed, better prediction results were obtained by 5-fold cross validation.

**Conclusions:**

An efficient method for predicting ion ligand binding sites was presented.

## Introduction

Ions play an important role in the structure and function of proteins: for example, the SO_4_^2−^ participate in the synthesis process of Cysteine [1], the sulfation process after protein translation [2], the synthesis process of proteoglycan, the sulfate absorption and decomposition process of plant and others [3]; the PO_4_^3−^ is an important component of bones and teeth which can maintain the neutrality of body fluids; alkali metal K^+^and Na^+^ control the charge balance in cells, tissue fluids and blood, which plays an important role in maintaining the normal circulation of body fluids and controlling the acid-base balance in the body; alkaline earth metal Ca^2+^ plays a regulatory role in nerve conduction and blood coagulation; transition metal Fe^3+^ plays an important role in the oxidative damage process of proteins, lipids, sugars and nucleic acids [4]. The interaction of proteins with ion ligands determines the realization of these biological functions, so the recognition of ion ligand binding sites is important for the study of its function [5–10].

In 2002, Richard et al. [11] have tested sulphate ion binding site of proteoglycan, and they identified the sites that is interaction with heparan sulfate. In 2017, Li et al. [12] used protein structural classification (SCOP) and Protein Data Bank (PDB) databases to extract 1251 protein chains using Ligand-Protein Contacts (LPC) software, and gave predictions of 8112 binding residues, and the Support vector machine (SVM) algorithm was used to predict the sulfate ion-binding residues of proteins. In recent years, the Zhang Lab team has compiled a database of ligand-binding residues named as the BioLip [13] database, a semi-manual database that collects interactions between ligands and proteins, functional annotations are relatively comprehensive compared with other databases, which contain extremely extensive and accurate ligand protein data.

During the last few years, many approaches have been developed to predict the binding sites of protein-metal ions. In 2008, Babr et al. [14] predicted the binding sites of protein chains and transition metal ions by CHED algorithm; when predicting 349 whole proteins, 95% specificity was obtained, and 82 prions were predicted to obtain 96% specificity. In 2012, Lu et al. [15] used the “fragment transformation” method to predict metal ion (Ca^2+^, Mg^2+^, Cu^2+^, Fe^3+^, Mn^2+^, Zn^2+^) ligand binding sites, and the prediction results were obtained with a total accuracy of 94.6% and a true positive of rate 60.5%. In 2016, Hu et al. [16] identified four metal ions in the BioLip database by both sequence-based and template-based methods, and the Matthew’s correlation coefficient (MCC) values were greater than 0.5. In 2017, Cao et al. [17] used the SVM algorithm to identify ten metal ion binding sites based on amino acid sequences, which obtained a good result by 5-fold cross validation. In 2018, Greenside et al. [18] used an interpretable confidence-rated boosting algorithm to predict protein-ligand interactions with high accuracy from ligand chemical substructures and protein 1D sequence motifs, which got a great result.

In this paper, the dataset of acid radical ion and metal ion ligands was extracted from BioLip database, the Sequential minimal optimization (SMO) algorithm was proposed to predict the binding site with component information, position conservation information and refinement characteristics, experiment results show that the MCC values of the four acid radical ion ligands by 5-fold cross validation exceeded 0.470, the accuracy values were not less than 74.0%; the MCC values of six metal ion ligands of Zn^2+,^ Cu^2+^, Fe^2+^, Fe^3+^, Mn^2+^ and Co^2+^ exceeded 0.620, the accuracy values were not less than 80%; the MCC values of four metal ions of Ca^2+^, Mg^2+^, Na^+^ and K^+^ exceeded 0.430, the accuracy values were not less than 71%.

## Materials and methods

### Dataset

The construction of the dataset is directly related to the reliability of the prediction accuracy. The dataset constructed in the paper was from the BioLip database.

The binding protein chains, including four acid radical ion ligands (NO^2−^, CO_3_^2−^, SO_4_^2−^,PO_4_^3−^) and ten metal ion ligands (Zn^2+^, Cu^2+^, Fe^2+^, Fe^3+^, Ca^2+^, Mg^2+^, Mn^2+^, Na^+^, K^+^,Co^2+^), were downloaded from the BioLip database, wherein the sequence length is greater than 50 residues, the resolution is less than 3 Å, and the sequence identity threshold is less than 30%. Then, the sliding window method is adopted to get the overlapping segment on the protein chain, if the center of the segment is the ligand binding site, it is defined as a positive sample; otherwise it is defined as a negative sample. We selected the datasets with the sequence segment length of 17 as an example to simply explain the multiple relationships of segments’ number in positive and negative sets; the detailed datasets are summarized in Table 1.

**Table 1:** Benchmark datasets of the sequence segment with length 17

Ion ligand	Protein chains	Positive set	Negative set
NO_2_^−^	22	98	8144
CO_3_^2−^	62	316	22,766
SO_4_^2−^	303	2125	99,729
PO_4_^3−^	339	2168	112,279
Zn^2+^	1428	6408	405,113
Cu^2+^	117	485	33,948
Fe^2+^	92	382	29,345
Fe^3+^	217	1057	68,829
Ca^2+^	1237	6789	396,957
Mg^2+^	1461	5212	480,307
Mn^2+^	459	2124	156,625
Na^+^	78	489	27,408
K^+^	57	535	18,777
Co^2+^	194	875	55,050

Since the number of samples in negative set is several tens of times the number of samples in positive set, in order to ensure stable of the results, the negative set with equal numbers of positive set was randomly selected ten times in the 5-fold cross validation, and finally the final result was obtained by selecting an average of ten times.

### The statistical analysis of dataset

#### Amino acid composition information

**Fig. 1:**
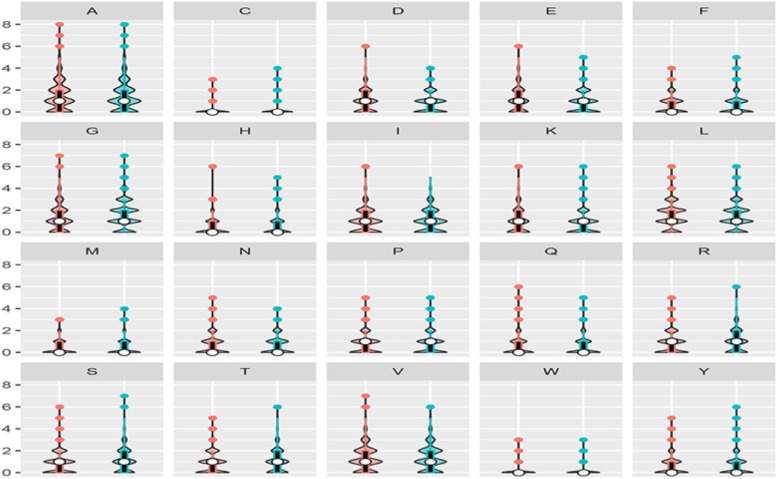
Violin plot of positive and negative segments of amino acid composition of SO_4_^2−^

According to the literature [12, 17], amino acid composition information is an important feature in the recognition of binding sites. Therefore, we analyzed the composition information of acid radical ion and metal ion ligand. The SO_4_^2−^ ligand was taken as an example, the violin plot was shown in Fig. 1. The violin plot is a combination of a box plot and a kernel density, and is mainly used to display the distribution state of the data. The left side of each group represents the amino acid composition in the negative set, the right side represents the amino acid composition in the positive set, the ordinate represents the frequency of occurrence of the amino acid, and the white dot represents the median. The black box pattern ranges from the lower quartile to the upper quartile, representing the concentrated distribution of amino acid; the outer shape represents the kernel density estimation, the more concentrated the data, the fatter the graph. Figure 1 showed that the concentrated distribution interval of R, S and T in the positive set was larger than the concentrated distribution of the negative set, while the D, E, G in the negative set were more concentrated than the positive set. Since the concentrated distribution interval of amino acid composition in the positive and negative sets was significantly different, we used the amino acid composition information as a characteristic parameter.

#### The position conservation of amino acids

**Fig. 2:**
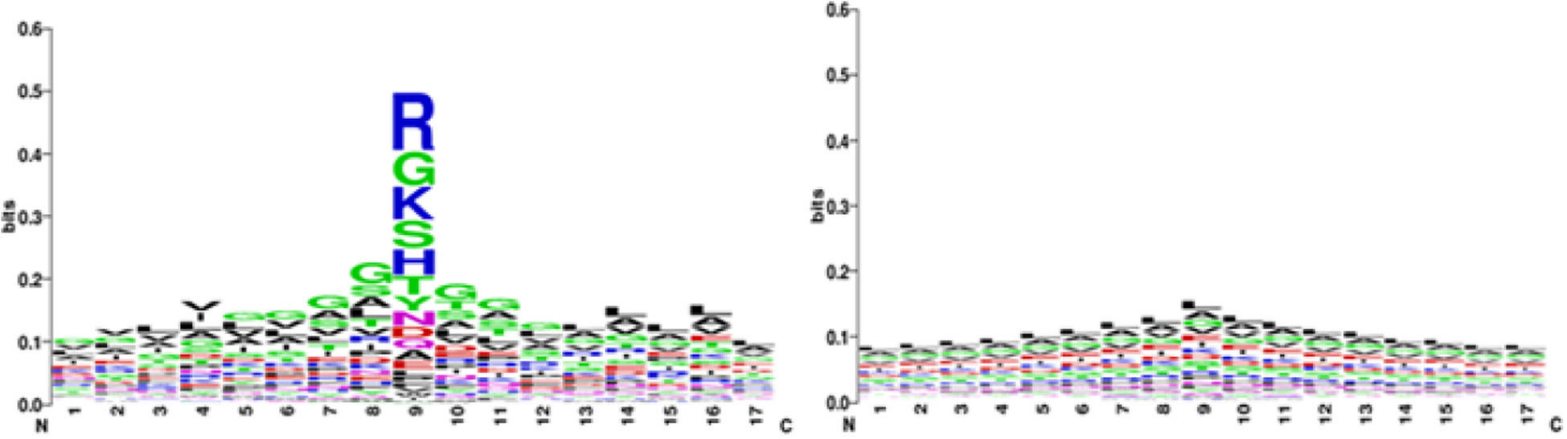
The position conservation of positive and negative amino acid in SO_4_^2−^. Note: the left figure indicates the position conservation in positive sequence segments and the right figure indicates the position conservation in negative sequence segments

The WEBLOGO [19] software was used to analyze the position conservation of acid radical ion and metal ion ligands. Since the ion ligands are small ligands, they usually only bind with a few residues. So we selected a window length *L* of 17 as an example to analyze. The x-axis represents 17 positions, the y-axis represents the conservation of amino acids in every position, with the height of each letter corresponding to the occurrence probability of the corresponding amino acid, the center of the positive set indicates the ion ligand binding residue. As shown in Fig.2, the position conservation of the SO_4_^2−^ binding residues and environmental residues are strong, but binding residues are more conservative, the preferred residues are R, G, K, S, H, T, and there is a significant difference of amino acid conservative between positive set and negative set. For example, at the eighth position, the highest frequency of the amino acid is G, S, A, L in positive set; the highest frequency of the amino acid in negative set is L, A, G, V. In the tenth positive, the highest frequency of amino acid is G, T, S, A in positive set; the highest frequency is L, A, G, V in negative set. The above analysis shows that the position conservation of amino acid residues is a good indicator of protein ion binding, so it was selected as the characteristic information to further develop an effective identification model.

### The selection of characteristic parameters

#### The characteristic parameters from statistical analysis

According to the statistical analysis of component information and position conservation information for amino acid, these two kinds of information were selected as characteristic parameters.

#### Physicochemical properties of amino acids

According to the biological background, the physicochemical properties of amino acid residues play an irreplaceable role in the binding of proteins to ions. Therefore, we chose the hydropathy and polarization charge of amino acids as characteristic parameters. The 20 amino acids are grouped into 6 kinds [20] according to hydropathy characteristic (Table 2) and 3 kinds [21] according to polarization charge: positive charged(K,R,P), negative charged(D,E), uncharged(N,Q,H,L,I,V,A,M,F,S,T,Y,W,C,G).

**Table 2:** The hydropathy characteristic of amino acid

Classification	Amino Acids	Classification	Amino Acids
strongly hydrophilic	R,D,E,N,Q,K,H	Proline	P
strongly hydrophobic	L,I,V,A,M,F	Glycine	G
weakly hydrophilic	S,T,Y,W	Cysteine	C

#### Predicted structural information

The prediction of secondary structure and solvent accessibility reflect the spatial structure information of the backbone and side chains [22], so we also extracted these information as characteristic parameters using ANGLOR [23] software. According to the predicted secondary structure information, the 20 amino acids are divided into 3 categories: α-helix, β-sheet and coil; according to the predicted relative solvent accessibility (SA), the 20 amino acids are divided into 2 categories: SA value is greater than 0.25 for exposure; SA value is less than 0.25 for burial.

#### The extraction of characteristic parameters

According to the statistical analysis, the component information of five characteristic parameters of amino acid, hydropathy, charge, secondary structure and relative solvent accessibility were selected, and the Increment of Diversity algorithm was used to reduce the dimension of the above five components to extract their refinement features; the Position matrix scoring algorithm was used to extract the site information of five characteristic parameters and reduce the dimension to extract their refinement features.

#### Position matrix scoring algorithm

The Position matrix scoring algorithm constructs a positional frequency matrix using known sequence patterns to describe the composition of amino acids at various positions in an unknown sequence pattern, and to characterize the position conservation of amino acids in the sequence. Through statistical analysis of the ion ligands in this study, it is found that they have obvious position conservation, so the Position matrix scoring algorithm was selected to extract the feature parameters.

Position matrix scoring algorithm is a classification algorithm. It has been successfully used in predicting transcription factor binding sites in genomes and super-secondary structures [24, 25].

The position frequency matrix is defined as:

1 $$ {p}_{i,j}=\frac{\left({n}_{i,j}+\frac{\sqrt{N_i}}{21}\right)}{\left({N}_i+\sqrt{N_i}\right)} $$

In the above equation, j is 20 amino acids and one pseudo amino acid “X”, *n*_*i*, *j*_ is the frequency of the j^th^ amino acids at the i^th^ position, *N*_*i*_ is total number of all amino acids occurring at the i^th^ position, *P*_*i,j*_ is the observed probability of the j^th^ amino acids at the i^th^ position.

The matrix element of the position weight matrix is defined as:

2 $$ {m}_{i,j}=\log \left(\frac{p_{i,j}}{p_{o,j}}\right)\kern6.75em $$

*P*_*0,j*_ is background probability of the j^th^ amino acid, *m*_*i,j*_ is the weight probability of the j^th^ amino acids at the i^th^ position.

The scoring(S) value is given by the following equation:

3 $$ \kern0.75em S=\frac{\sum \limits_{i=1}^L{C}_i\left({m}_{i,j}-{m}_{i,\min}\right)}{\sum \limits_{i=1}^L{C}_i\left({m}_{i,\max }-{m}_{i,\min}\right)}\kern3.25em $$

Here,

4 $$ {C}_i=\frac{100}{\log 21}\left(\sum \limits_{i=1}^{21}{p}_{i,j}\log {p}_{i,j}+\log 21\right) $$

S is the scoring matrix function, L is length of amino acid sequence segment, *C*_*i*_ is conservation index at the i-th position, *m*_*i,min*_ is the minimum value at the i^th^ position, *m*_*i,max*_ is the maximum value at the i^th^ position.

Taking the position amino acid residue as a parameter, two standard scoring matrices were constructed using the training set. In the test set, two scoring (S) values can be obtained for an arbitrary sequence segment, which can be used as the refinement characteristic parameters. Besides, the characteristic parameters of the 2 L dimensional site information can also be obtained by using the position weight matrix.

#### Increment of diversity (ID) algorithm

Dispersion is a measure of information diversity. It can quantitatively describe certain feature information contained in an amino acid sequence, and the measure of diversity can describe the overall diversity. The increment of diversity is one of the information coefficients. It is applied to the information classification as a classification algorithm. It can reduce the dimension and use the refined features as the characteristic parameters of classification prediction. It has been successfully applied to protein folding and protein structure classification prediction [26, 27]. Therefore, the Increment of Diversity algorithm was used to extract the feature information from sequence.

In the state space of dimension S, for a vector *X*: [*n*_*1,*_ *n*_*2*_*, …,n*_*s*_] the measure of diversity source was

5 $$ D(X)=N\log N-\sum \limits_{i=1}^s{n}_i\log {n}_i $$

For two state spaces of dimension S, for vectors *X*: [*n*_*1,*_ *n*_*2*_*, … n*_*s*_] and *Y*: [*m*_*1*_*, m*_*2*_*, …, m*_*s*_], the measure of mixed diversity source *X* + *Y* was

6 $$ D\left(X,Y\right)=\left(N+M\right)\log \left(N+M\right)-\sum \limits_{i=1}^s\left({n}_i+{m}_i\right)\log \left({n}_i+{m}_i\right) $$

The increment of diversity between the source of diversity *X* and *Y* was

7 $$ ID\left(X,Y\right)=D\left(X+Y\right)-D(X)-D(Y) $$

The amino acid composition information was input into the ID algorithm. The standard discrete source is constructed by training. Two discrete increment (ID) values can be obtained for each segment of the test set. Then, the obtained two-dimensional ID value can be used as the characteristic parameter.

#### Algorithm

The SMO algorithm was proposed by Platt in 1998, which is also known as the sequence minimum optimization method. It is the fastest quadratic programming optimization algorithm that can effectively improve computational efficiency. The SMO algorithm optimizes only two variables at a time, regards all other variables as constants, transforms a complex optimization problem into a relatively simple two-variable optimization problem, and adopts analytical method to avoid the error accumulation caused by iteration method, which ensures its accuracy. In this paper, we established our identification model using the SMO algorithm based on the weka3.8 [28, 29] and using the Precomputed Kernel Matrix (PUK) kernel function. PUK is a general kernel function based on Pearson’s seventh function [30]. It has good robustness and has equivalent or even stronger mapping ability than standard kernel functions. It can be used as a general kernel function to replace ordinary linear, polynomial and radial basis kernel functions. To a certain extent, it can eliminate the trouble of how to select the kernel function in the SVM algorithm, saving time.

#### Performance measure

We used the following four standard measures [31] to evaluate the performance of the identification of ion binding residues: sensitivity (S_n_), specificity (S_p_), accuracy of prediction (Acc) and Matthew’s correlation coefficient (MCC). These were calculated by the following formulae:

8 $$ {S}_n=\frac{TP}{TP+ FN}\times 100\% $$

9 $$ {S}_p=\frac{TN}{TN+ FP}\times 100\% $$

10 $$ Acc=\frac{TP+ TN}{TP+ TN+ FP+ FN}\times 100\% $$

11 $$ MCC=\frac{\left( TP\times TN\right)-\left( FP\times FN\right)}{\sqrt{\left( TP+ FP\right)\left( TP+ FN\right)\left( TN+ FP\right)\left( TN+ FN\right)}} $$

Where TP is the number of correctly identified acid radical or metal ion binding residues, FN is the number of binding residues wrongly identified as non-binding residues, TN is the number of correctly identified non-binding residues, and FP is the number of non-binding residues identified as binding residues.

## Results and discussion

### The optimal window size

**Fig. 3:**
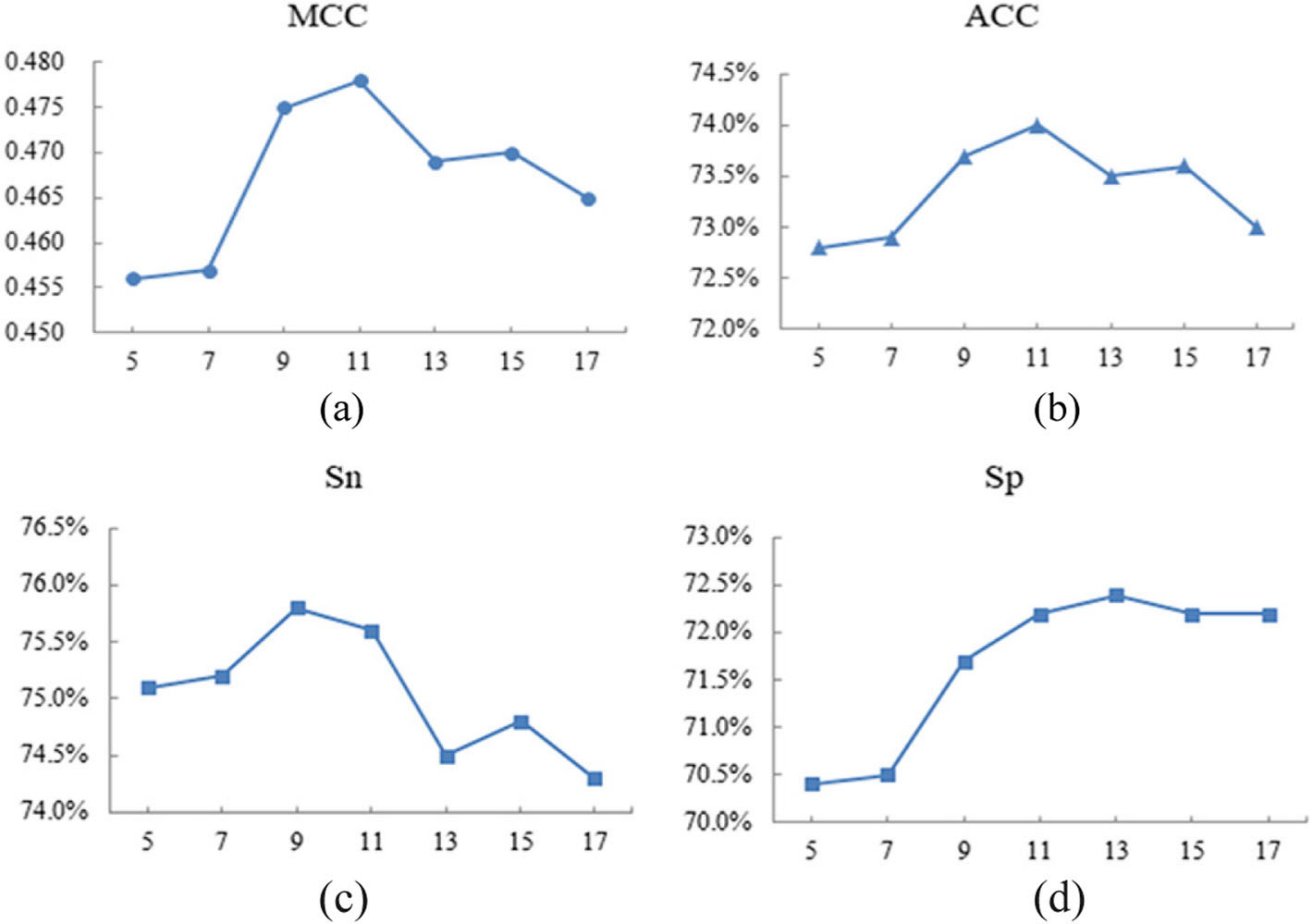
The results of SO_4_^2−^‘s evaluation index under different window sizes. Note: (**a**) MCC values of SO_4_^2−^ under different window sizes; (**b**) Acc values of SO_4_^2−^ under different window sizes; (**c**) S_n_ values of SO_4_^2−^ under different window sizes; (**d**) S_p_ values of SO_4_^2−^ under different window sizes

Whether the amino acid residue can be combined with the ion ligand depends not only on amino acid residue itself but also on neighboring residues [32]. In order to extract more comprehensive information, we used the sliding window method, where different window sizes range from 5 to 17, intercepting the sequence segments from the N-terminal to the C-terminal, and ensuring that all residues appear in the center of the segment, we added an (L-1)/2 dummy residue “X” at both terminals of the proteins. If the central residue of the segment was an ion binding residue, we assigned the segment as positive; otherwise it was assigned as negative. Taking SO_4_^2−^ ligand as an example (Fig. 3), the x-axis represents the window size, the y-axis represents the MCC, ACC, S_n_ and S_p_ values under different window sizes, we performed a large range search on the window size of 7 kinds of amino acid residues and combined the WEBLOGO diagram of the ion ligand to finally determine the optimal window size of SO_4_^2−^ is 11, other ion ligand of NO_2_^−^, CO_3_^2−^, PO_4_^3−^, Zn^2+^, Cu^2+^, Fe^2+^, Fe^3+^, Ca^2+^, Mg^2+^, Mn^2+^, Na^+^, K^+^ and Co^2+^ are: 11, 13, 9, 7, 13, 9, 9, 9, 9, 7, 9, 11, 11.

The following calculations were made under the optimal window sizes and the 5-fold cross validation commonly used in the literature [33–35].

### The results under component information parameters

Under the optimal window size, amino acid component information, hydropathy component information, charge component information, secondary structure component information, and relative solvent accessibility component information were collectively used as characteristic parameters and input to the SMO algorithm. The calculation results of 5-fold cross validation were shown in Table 3.

**Table 3:** Recognition results of ion binding sites based on component information

Ligand	S_n_ (%)	S_p_ (%)	Acc (%)	MCC
NO_2_^−^	65.3	57.1	61.2	0.225
CO_3_^2−^	68.7	67.7	68.2	0.364
SO_4_^2−^	68.9	67.2	68.0	0.360
PO_4_^3−^	67.8	63.8	65.8	0.435
Zn^2+^	75.9	81.6	78.8	0.576
Cu^2+^	73.8	80.6	77.2	0.546
Fe^2+^	77.0	80.1	78.5	0.571
Fe^3+^	75.1	78.1	76.6	0.533
Ca^2+^	68.1	72.5	70.3	0.406
Mg^2+^	66.7	74.8	70.8	0.417
Mn^2+^	72.5	77.3	74.9	0.498
Na^+^	72.6	70.8	71.7	0.434
K^+^	75.0	75.7	75.3	0.507
Co^2+^	71.7	74.3	73.0	0.460

It can be observed from Table 3 that the ACC values of the four acid radical ion ligands were all greater than 61.0%, the MCC values of CO_3_^2−^, SO_4_^2−^ and PO_4_^3−^ exceed 0.360, and only the MCC value of NO_2_^−^ was lower than 0.225; among the recognition results of metal ion ligands, Zn^2+^, Cu^2+^, Fe^2+^, Fe^3+^ and K^+^ were preferable, and the MCC values were not less than 0.5. It can be considered that these five metal ion ligands were sensitive to the component information; the results were consistent with the previous research results. The reason can be seen from the statistical diagram of the amino acid composition given in [17] that the differences of positive and negative sets of transition metal ions were relatively large, so their prediction results were better, and the remaining ion ligands will continue to be identified by adding other characteristic parameters.

### The results under position conservation information parameters

Under the optimal window size, we identified the ion ligand binding sites using position amino acid, position hydropathy, position charge, position secondary structure and position relative solvent accessibility as characteristic parameters via the SMO algorithm. The calculation results by 5-fold cross validation were shown in Table 4.

**Table 4:** Recognition results of ion binding sites based on position conservation information

Ligand	S_n_ (%)	S_p_ (%)	Acc (%)	MCC
NO_2_^−^	73.5	61.2	67.4	0.350
CO_3_^2−^	67.4	78.5	72.9	0.462
SO_4_^2−^	74.2	71.8	73.0	0.460
PO_4_^3−^	75.3	79.4	77.4	0.548
Zn^2+^	91.6	87.2	89.4	0.789
Cu^2+^	84.5	88.9	86.7	0.735
Fe^2+^	90.1	79.6	84.8	0.700
Fe^3+^	80.9	85.7	83.3	0.667
Ca^2+^	69.4	75.7	72.5	0.451
Mg^2+^	69.5	76.5	73.0	0.461
Mn^2+^	78.1	84.4	81.2	0.626
Na^+^	65.8	73.2	69.5	0.392
K^+^	73.8	58.7	66.3	0.329
Co^2+^	75.7	84.1	79.9	0.600

From Table 4, it can be concluded that the MCC value of NO_2_^−^ was 0.350, the MCC value of CO_3_^2−^ was 0.462, the MCC value of SO_4_^2−^ was 0.460, and the MCC value of PO_4_^3−^ was 0.548. Compared with all component information as characteristic parameters, the recognition result has been improved.

For the identification results of ten metal ion ligands, the six metal ion ligands of Zn^2+^, Cu^2+^, Fe^2+^, Fe^3+^, Mn^2+^ and Co^2+^ have good prediction results, and the MCC values were not less than 0.600; Na^+^ and K^+^ have worst recognition results, we considered that these two ion ligands were less sensitive to the position conservation information and can continue to identify their refinement. Compared with the identification of all the component information as characteristic parameters, the MCC values of Na^+^ and K^+^ decreased slightly, but other’s MCC values showed an upward trend, indicating that these ion ligands were more sensitive to the position conservation information, as can be seen from the WEBLOGO in [17]. The positive and negative sets are more different than the statistical analysis of the components in [17], so the ion ligands were more sensitive to the position conservation information.

### The results under refinement characteristic parameters

The ID algorithm was used to reduce the dimensionality of the amino acid component information, hydropathy component information, charge component information, secondary structure component information, and relative solvent accessibility component information to obtain a 10-dimensional ID value; the Position matrix scoring algorithm reduced the dimensionality of the position amino acid, position hydropathy, position charge, position secondary structure and position relative solvent accessibility to obtain a 10-dimensional S value. The obtained 10-dimensional ID value and 10-dimensional S value were collectively recognized as the 20-dimensional refinement characteristic by the SMO algorithm, and the results (OUR’S) by 5-fold cross validation were shown in Table 5.

**Table 5:** Comparison results with SVM

Ligand	Method	S_n_ (%)	S_p_ (%)	Acc (%)	MCC
Zn^2+^	OUR’S	94.2	84.2	89.2	0.789
	SMO	94.8	83.7	89.3	0.790
	SVM	99.8	99.5	99.7	0.993
Cu^2+^	OUR’S	91.3	86.8	89.0	0.782
	SMO	90.3	88.9	89.6	0.792
	SVM	95.5	97.1	96.3	0.926
Fe^2+^	OUR’S	90.1	81.9	86.0	0.722
	SMO	89.3	82.5	85.9	0.719
	SVM	91.9	90.7	91.3	0.826
Fe^3+^	OUR’S	86.2	85.5	85.9	0.717
	SMO	85.5	86.0	85.8	0.715
	SVM	86.9	88.7	87.8	0.756
Ca^2+^	OUR’S	68.8	75.3	72.1	0.443
	SMO	69.5	75.4	72.5	0.450
	SVM	71.3	79.1	74.8	0.502
Mg^2+^	OUR’S	71.1	73.1	72.1	0.442
	SMO	70.0	72.3	71.2	0.423
	SVM	76.6	73.9	75.3	0.505
Mn^2+^	OUR’S	82.0	83.9	83.0	0.659
	SMO	80.3	83.3	81.8	0.636
	SVM	82.1	84.4	83.2	0.664
Na^+^	OUR’S	68.9	74.0	71.0	0.430
	SMO	70.8	71.8	71.3	0.425
	SVM	82.2	76.2	79.4	0.586
K^+^	OUR’S	71.6	64.5	68.0	0.362
	SMO	74.2	62.6	68.4	0.371
	SVM	77.3	83.2	80.3	0.607
Co^2+^	OUR’S	75.3	86.4	80.9	0.621
	SMO	75.1	86.2	80.6	0.616
	SVM	80.8	85.1	83.0	0.660
NO_2_^−^	OUR’S	80.6	88.8	84.7	0.696
	SMO	–	–	–	–
	SVM	–	–	–	–
CO_3_^2−^	OUR’S	79.4	81.6	80.5	0.611
	SMO	–	–	–	–
	SVM	–	–	–	–
SO_4_^2−^	OUR’S	75.6	72.2	74.0	0.478
	SMO	–	–	–	–
	SVM	–	–	–	–
PO_4_^3−^	OUR’S	76.2	78.0	77.1	0.542
	SMO	–	–	–	–
	SVM	–	–	–	–

At the same time, for the sake of comparison, the results of the SVM algorithm in paper [17] and the calculation results of SMO using the characteristic parameters of literature [17] were also included in Table 5.

As seen, the four acid radical ion ligands under the refinement characteristic parameters were very good, the MCC values were over 0.460, and the Acc values were all greater than 73.0%. Compared with the recognition results of all component information and all position conservation information, the values of S_n_, S_p_ and Acc were gradually improved, indicating that the detailed characteristic parameters contain more complete information.

The MCC values of Zn^2+^, Fe^2+^, Fe^3+^and Cu^2+^ have reached above 0.7, the MCC values of Mn^2+^and Co^2+^ exceed 0.6, and the MCC value of K^+^ was only 0.362; the MCC values of the eight metal ion ligands of Zn^2+^, Cu^2+^, Fe^2+^, Fe^3+^, Mn^2+^, Na^+^, K^+^ and Co^2+^ were improved in a small range compared with the results in Table 4, indicating that the eight ion ligands were more sensitive to the refinement characteristic; the evaluation indexes of Ca^2+^ and Mg^2+^with the refinement characteristic parameters were not higher than that with the position conservation information, indicating that these two ion ligands were more sensitive to position conservation information; the Na^+^ and K^+^ have higher MCC values when the refinement characteristic was used as a parameter, compared with the results of all component information as characteristic parameters, it can be understood that Na^+^ and K^+^ were more sensitive to all component information under three characteristic parameters, but still lower than the results of other metal ion ligands, the MCC values of the residual ion ligands under the refinement characteristic parameters were improved compared with the results of all component information, which was the best results under the three characteristic parameters.

In general, the recognition result under the refined characteristic parameters was generally higher than the recognition result under the single combination characteristic parameter, which fully demonstrated that the compatibility performance of the SMO algorithm is good.

### Comparison with the results of SVM

The data showed that although the results under the SVM algorithm were better overall than those under the SMO algorithm, their overall prediction trends were the same. The prediction results of individual ions were close to those of SVM. For example, Mn^2+^, the MCC value reached 0.663 under SVM algorithm, and the MCC value reached 0.636 under SMO algorithm.

In addition, new characteristic parameters were added based on the SMO results, and the prediction results for some ion ligands were improved, that is, the results of OUR’S in Table 5, indicating that the new characteristic parameters we added were useful parameters, suitable for the SMO algorithm.

Overall, in the process of ion ligand binding sites prediction, the SMO algorithm adopts analytical method to avoid the error accumulation caused by iteration method, so the accuracy of the prediction result is guaranteed; the PUK kernel function of this algorithm can deal with the nonlinear classification data of the binding sites prediction well and reflect the distribution characteristics of the training sample data, since it maps features from low-dimensional space to high-dimensional space, and achieves linear separability. Therefore, the SMO algorithm has a good performance for the prediction of ion ligands.

## Conclusion

In this paper, the ligand binding sites of four acid radical ions and ten metal ions were predicted. Firstly, BioLip database was selected, and the optimal window sizes were determined by calculation; secondly, component information, position conservative information and detailed characteristics were extracted as characteristic parameters; then different characteristic parameters were input into the SMO algorithm, under the 5-fold cross validation, the identification of four kinds of acid radical ion ligand binding sites got a good result, among the results of the identification of ten metal ion ligands, the prediction results of transition metals were better than those of alkaline earth metals and alkali metals, the results of all position conservation information as characteristic parameters were better than the results of all component information as characteristic parameters, the prediction results under the refinement characteristic were better than the prediction results under the single combination characteristic, so the characteristic parameters can be refined as much as possible in the subsequent work.

## Data Availability

If you need data and materials, you can contact the corresponding author**.**

## Abbreviations

Acc: Accuracy of prediction
ID: Increment of Diversity
LPC: Ligand-Protein Contacts
MCC: Matthew’s correlation coefficient
PDB: Protein Data Bank
PUK: Precomputed Kernel Matrix
S: scoring
SA: Relative solvent accessibility
SCOP: Protein structural classification
SMO: Sequential minimal optimization
S_n_: Sensitivity
S_p_: Specificity
SVM: Support vector machine

## References

[1] Leustek T, Murillo M, Cervantes M (1994). Cloning of a cDNA encoding ATP sulfurylase from Arabidopsis thaliana by functional expression in Saccharomyces cerevisiae [J]. Plant Physiol.

[2] Monigatti F, Gasteiger E, Bairoch A (2002). The Sulfinator: predicting tyrosine sulfation sites in protein sequences [J]. Bioinformatics.

[3] Hatzfeld Y, Lee S, Lee M (2000). Functional characterization of a gene encoding a fourth ATP sulfurylase isoform from Arabidopsis thaliana [J]. Gene.

[4] Lv X, Tan X (2013). Metals homeostasis and related proteins in Alzheimer's disease [J]. Progress in Chemistry.

[5] Bao W, Jiang Z, Huang DS (2017). Novel human microbe-disease association prediction using network consistency projection [J]. BMC Bioinformatics.

[6] Deng SP, Cao S, Huang DS (2017). Identifying stages of kidney renal cell carcinoma by combining gene expression and DNA methylation data [J]. IEEE/ACM Trans Comput Biol Bioinform.

[7] Guo W, Zhu L, Deng S, et al. Understanding tissue-specificity with human tissue-specific regulatory networks [J]. Science China Inf Sci. 2016;59(7):070105.

[8] Deng Su-Ping, Zhu Lin, Huang De-Shuang (2016). Predicting Hub Genes Associated with Cervical Cancer through Gene Co-Expression Networks. IEEE/ACM Transactions on Computational Biology and Bioinformatics.

[9] Deng SP, Zhu L, Huang DS (2015). Mining the bladder cancer-associated genes by an integrated strategy for the construction and analysis of differential co-expression networks [J]. BMC Genomics.

[10] Huang DS, Zheng CH (2006). Independent component analysis-based penalized discriminant method for tumor classification using gene expression data [J]. Bioinformatics.

[11] Warner RG, Hundt C, Weiss S (2002). Identification of the heparan sulfate binding sites in the cellular prion protein [J]. J Biol Chem.

[12] Li S, Hu X (2017). Identifying the sulfate ion binding residues in proteins [J].

[13] Yang J, Roy A, Zhang Y (2013). BioLiP: a semi-manually curated database for biologically relevant ligand-protein interactions [J]. Nucleic Acids Res.

[14] Sobolev V, Edelman M (2013). Web tools for predicting metal binding sites in proteins [J]. Israel J Chemistry.

[15] Lu CH, Lin YF, Lin JJ (2012). Prediction of metal ion–binding sites in proteins using the fragment transformation method [J]. PLoS One.

[16] Hu X, Wang K, Dong Q (2016). Protein ligand-specific binding residue predictions by an ensemble classifier [J]. BMC Bioinformatics.

[17] Cao X, Hu X, Zhang X (2017). Identification of metal ion binding sites based on amino acid sequences [J]. PLoS One.

[18] Greenside P, Hillenmeyer M, Kundaje A (2018). Prediction of protein-ligand interactions from paired protein sequence motifs and ligand substructures. Pacific symposium.

[19] Liu T, Lin Y, Wen X (2007). BindingDB: a web-accessible database of experimentally determined protein-ligand binding affinities [J]. Nucleic Acids Res.

[20] Panek J, Eidhammer IR (2005). A new method for identification of protein (sub) families in a set of proteins based on hydropathy distribution in proteins [J]. Proteins-structure Funct Bioinformatics.

[21] Taylor WR (1986). The classification of amino acid conservation.[J]. J Theor Biol.

[22] Chen H (2005). Prediction of solvent accessibility and sites of deleterious mutations from protein sequence [J]. Nucleic Acids Res.

[23] Wu S, Zhang Y (2008). ANGLOR: a composite machine-learning algorithm for protein backbone torsion angle prediction [J]. PLoS One.

[24] Kel AE (2003). E. Gößling, Reuter I, et al. MATCHTM: a tool for searching transcription factor binding sites in DNA sequences [J]. Nucleic Acids Res.

[25] Hu X, Li Q (2010). Using support vector machine to predict - and -turns in proteins [J]. J Comput Chem.

[26] Zhenxing F, Xiuzhen H (2014). Recognition of 27-class protein folds by adding the interaction of segments and motif information [J]. Biomed Res Int.

[27] Lei L, Xiuzhen H (2012). Predicting protein fold types by the general form of Chou’s pseudo amino acid composition: approached from optimal feature extractions [J]. Protein Pept Lett.

[28] Feng ZX, Li QZ (2017). Recognition of long-range enhancer-promoter interactions by adding genomic signatures of segmented regulatory regions [J]. Genomics.

[29] Hall M, Frank E, Holmes G, Pfahringer B, Reutemann P, Witten IH (2009). The WEKA data mining software: an update. ACM SIGKDD Explor Newsl.

[30] Üstün B, Melssen W, Buydens L (2006). Facilitating the application of support vector regression by using a universal Pearson VII function based kernel [J]. Chemometrics Intell Lab Syst.

[31] Sun T, Zhou B, Lai L (2017). Sequence-based prediction of protein protein interaction using a deep-learning algorithm [J]. Bioinformatics.

[32] Jiang Z, Hu XZ, Geriletu G, et al. Identification of Ca^2+^-binding residues of a protein from its primary sequence [J]. Genet Mol Res. 2016;15(2):gmr.15027618. 10.4238/gmr.15027618 27323050

[33] Hu X, Dong Q, Yang J (2016). Recognizing metal and acid radical ion-binding sites by integrating ab initio modeling with template-based transferals [J]. Bioinformatics.

[34] Wang Tao, Li Liping, Huang Yu-An, Zhang Hui, Ma Yahong, Zhou Xing (2018). Prediction of Protein-Protein Interactions from Amino Acid Sequences Based on Continuous and Discrete Wavelet Transform Features. Molecules.

[35] Yi Hai-Cheng, You Zhu-Hong, Huang De-Shuang, Li Xiao, Jiang Tong-Hai, Li Li-Ping (2018). A Deep Learning Framework for Robust and Accurate Prediction of ncRNA-Protein Interactions Using Evolutionary Information. Molecular Therapy - Nucleic Acids.

